# Stem Cells and Neuroprotection: Understanding the Players

**DOI:** 10.3390/ijms11093288

**Published:** 2010-09-15

**Authors:** Virginia Pearce

**Affiliations:** Department of Molecular Biology and Immunology, University of North Texas Health Science Center, Fort Worth, TX 76011, USA; E-Mail: virginia.pearce@unthsc.edu; Tel.: +1-817-735-0617; Fax: +1-817 735-2118

**Keywords:** stem cells, neuroprotection, gender specific, telomerase, mitochondria, renewal

## Abstract

The use of neuroprotective therapies begs the question of how such therapies could affect preexisting stem cell populations within the host, as well as those introduced through cell-replacement therapy. Multiple mechanisms may mediate stem cell responses to neuroprotectants such as host/donor age and gender, cellular lineage/differentiation status, and mitochondrial dynamics. Current therapeutic sources for stem cells are embryonic, somatic, or induced pluripotent, with very little known about the effects of gender, age, cell type, and mitochondrial dynamics. With the advent of therapies to stimulate and recruit endogenous stem cells or transplant donor cells into damage areas in the hopes of recuperative regeneration of lost neurons, it is important to discuss mechanisms that dictate the winning players in the neuroprotection game. This review will focus on our current understanding of the characteristics of renewing stem cells that may affect neuroprotection.

## 1. Introduction

The potential impact of stem cell research was declared on the cover of Time magazine as the “coming revolution in stem cells” [1]. With new administrative initiatives and NIH guidelines in place, the race is on to establish new and valid stem cell lines for new cures. Fueling the revolution are billions of dollars in stem cells initiatives that over time will demand a return on investments [2]. So, it is important that we get it right. To do so, we must know the character and function of the stem cells used in treatment protocols. As so apply stated, “…it will not be the cells themselves that will be so important, but rather what we learn about them and the way they function.” [3]. This review will touch on our expanding knowledge of certain less emphasized aspects of the stem cell character such as host/donor age and gender, cellular lineage/differentiation status, telomerase, and mitochondrial dynamics. While knowledge of mitochondrial dynamics in renewing cells is expanding, less is known about how telomerase, age, and gender can influence therapeutic responses, dictate and/or modulate neuroprotective responses. Further, we know very little about how these characters interact and become diverse factors that induce alterations in stem cells or modulate their environment during renewal. The same stem cell characteristics that we seek to use to our advantage in neuroprotective treatments are the characteristics that can mediate disease states. Therefore, it is important that we recognize all stem cell characteristics and functions that could impact treatments, as well as serve to improve treatment protocols aimed at either preventing neurodegeneration or inducing neurogenesis.

## 2. Multiple Mechanisms May Mediate Stem Cell Responses

### 2.1. Stem Cell Types and Renewal

A goal of neuroprotection is to restore or facilitate replacement of normal cell function, tissue, and/or organ due to damage [4]. Neural stem cells provide a renewable source of replacement cells, and increased proliferation of neural stem cells can act as a protective mechanism. Cells that renew themselves and ultimately differentiate do so through cycles of asymmetrical and symmetrical divisions that ultimately creates a hierarchy of various cell types. These cells types can be derived from adult stem cells (ASC), tissue specific progenitor cells, somatic cell nuclear transfer (SCNT) cells, single cell embryo biopsy, arrested embryos, altered nuclear transfer cells, reprogramming somatic cells (IPS), and embryonic stem cells (ESC) [4]. Each source has advantages and limitations mediated by genetic and/or epigenetic factors [5]. More specifically, the advantages include pluripotency in ESC where cells can differentiate into all germ layers, multipotency in ASC where cells can differentiate into limited lineages, *versus* transdifferentiation where cells can differentiate into a different lineage than its original state (SCNT, IPS) [6]. However, advantages associated with plasticity, can become disadvantages associated with unlimited growth and tumor-forming potential [5].

**Figure 1:**
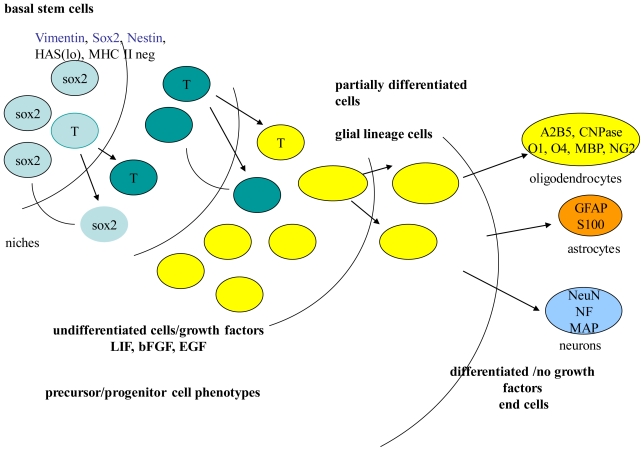
Replacement cells are provided through a hierarchy of cell types that result when self-renewing stem cells proliferate through asymmetrical and/or symmetrical division to form precursor and progenitor cells. Upon differentiation, neurons are formed.

The mammalian central nervous system (CNS) contains a hierarchy of self-renewing cells that proliferate, and are multipotent for neuronal and glial sub-types, that then differentiate into lineage cells (precursors or progenitors). Lineage-specific precursors or progenitors include neuronal, astroglial, glial, and oligodendroglial [7]. Cell types, within the stem cell hierarchy that include precursors and progenitors, can be identified by certain proteins characteristically expressed during renewal and differentiation (Figure 1). These characteristic proteins can influence cell fate and survival under certain environmental conditions or niche within regions of the brain [8]. Similarly, ESC are identified by certain expressed proteins that are not characteristic of ASC. More recently, microRNAs (miRNAs) profiles identified in ESC demonstrate gene expression patterns involved in renewal and/or differentiation [9–11]. Further, such profiles may be sex-specific during early stages of differentiation [12].

**Figure 2:**
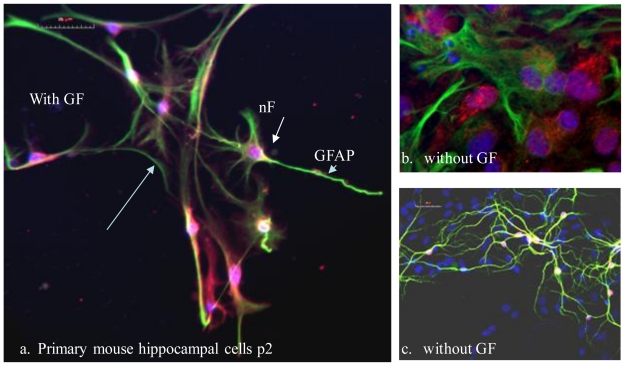
Primary mouse hippocampal cells cultured *in vitro* on coverslips with (a) and without growth factors (GF) (b, c), then fixed and stained for neuronal cells with markers neurofilament (nF) red and glial cells with (GFAP) green (a, b), and neural cells MAP2 (c) with DAPI stained nuclei (blue).

Acute neurological insults stimulate a basal rate of neural progenitor/precursor proliferation and differentiation [7,13]. This ability to repair damage due to loss of neurons may be dictated by the available neuronal precursor/progenitor and their mitogenic factors before repair can proceed [14]. In the hippocampus, ASC give rise to both glial and neurons through hippocampal precursors [15,16]. Mitogenic factors such as epidermal growth factor (EGF) promote proliferation of hippocampal precursors in serum free media, and aid in promoting neural stem cell renewal [7,17]. EGF acts through its respective receptor (EGFR) to induce receptor phosphorylation, then initiate intracellular signaling pathways that are involved in cell growth and physiology [18]. Studies indicate that EGF promotes neurogenesis/self repair, and correspondingly EGFRs are unregulated after injury [13]. *In vitro* studies using primary hippocampal cultures provide relevant characterization of cellular composition and mitogenic growth conditions that affect proliferation and/or differentiation during hippocampal renewal (Figure 2) [7,14,21].

### 2.2. Stem Cells Age

Stem and progenitor cells’ proliferation potential decreases with age, and thereby make them susceptible to enhanced oxidative stress and accumulation of mutations [22]. Based more on rodent studies than human, the frequency and rate of neurogenesis as well as self-renewal and mitotic potential of neural stem/progenitor cells in subventricular and subgranular zones decline with age [23–25].

ESC, FSC, and young and old ASC are maintained by differing renewal programs that change over time in response to tissue growth, damage, and repair [26]. In addition, declining trophic/growth factors may play a role in age related loss of stem cells [14]. Li *et al*. propose that stem cell aging may be heterogeneous among individuals, with some individuals having an advantage over aging with any/or all aspects of sustained neurogenesis, promotion of differentiation, enhancement of proliferation, and facilitation of neural plasticity [27]. It was suggested by studies using mice generated neurospheres that aging may lead to mutational load within the stem cell compartment [28].

### 2.3. Telomerase “Immortality”

An important factor related to neural stem cell aging is telomerase activity. Telomerase is active during embryogenesis and therefore active in ESC. It becomes inactive in postnatal somatic tissues, but maintains low activity in renewing basal ASC found in the subgranular zone of the hippocampal and dentate gyrus [29]. During renewal, telomere maintenance may be essential for prolonged stem cell function [30–32]. While telomerase is important in maintaining chromosomal integrity and cell viability in these stem-cell reserves, it cannot maintain telomeres. Therefore, telomeres will continue to shorten during ageing and/or replicative stress [25,29,33]. During neurogenesis, telomerase is active in renewing stem cells, down regulated during differentiation, and if overexpressed inhibits differentiation [34]. All of these studies beg the question of what happens with telomerase in IPS and/or SCNT cells, and does the age of donor matter? Will we need to screen donors for telomere length to optimize our stem cells? If we know our donor has short telomeres does that make them a poor donor and would that be similar to transplanting a damaged heart?

Human population studies show telomere maintenance and telomerase length or reserve is important in healthy lifespan and is linked with better cognitive function [25]. Studies in telomerase knockout mice showed that neural stem cell renewal and differentiation was compromised in aged mice [25]. Further, studies of ageing syndromes indicate association with telomere dysfunction and that stem cells may be more sensitive to telomere dysfunction based on cell type and environment [25,29]. Through telomerase dysfunction, ageing stem cells can become susceptible to transformation. Add to all this, genomic integrity as guarded by p53, which has two divergent aspects, pro- or anti-aging dependent on stem cell type and telomerase dysfunction [25]. Further, hTERT the catalytic subunit of telomerase, can affect stem cell function independent of telomere maintenance in an age-relevant manner [29]. These complex factors which we are only being to elucidate and understand make telomere dysfunction a major factor in limiting stem cell function and engraftment [29].

### 2.4. “Gender Matters”

An overlooked variable in stem cell biology, gender is an important donor factor when considering transplantation [35,36]. Gender differences in donor stem cells is also an important factor in neuroprotection, though conclusions on sexual dimorphism mediated neuroprotection vary. These varying conclusions dictate more understanding of molecular and cell-based mechanisms when determining host gender differences regarding neuroprotection [37–40]. Differing stem cell transplant studies in both human and rodent, indicate sex dimorphism in stem cells used in transplants [41]. Yuan *et al.* when working with bone marrow mesenchymal stem cells proposed the need for more cellular biology studies relating to underlying gender differences and their potential role in neurogenesis [42]. Ongoing studies indicate gonadal hormones can affect neurogenesis in a gender specific manner by influencing growth and survival [43]. Some studies suggest a general female advantage applicable to stem cell transplants including potency, survival, and secretory patterns that may be more than hormone mediated [41]. Bianchi and Fisk raised the issue of microchimerism for consideration when the stem cell donor or recipient is a woman who has been pregnant [44]. This phenomenon also referred to as transplacental feto-maternal cell trafficking, occurs in post pregnant women who retain persistent and long-term fetal cells within their bodies either after delivery or termination [44–46]. Further, this biological concept is the ultimate gender matters factor relating to clinical application for stem cell therapies. Gender differences create both multiple and complex parameters that need to be defined and understood before we can influence stem cell proliferation and survival during neurogenesis [41,45].

### 2.5. Mitochondrial Dynamics

**Figure 3:**
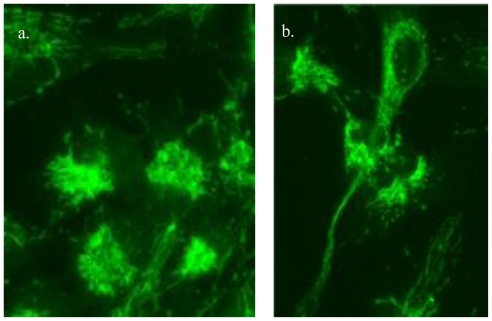
Human neural stem cells showing mitochondria (green) in progenitors with (a) perinuclear staining, and (b) more cytoplasmic staining.

As the powerhouse of the cell, mitochondria are responsible for aerobic respiration and essential for steroid metabolism, calcium homeostasis, apoptosis, and cellular proliferation [47]. Stem cells contain few mitochondria that are heterogeneous between stem cell types. Mitochondria properties including subcellular and metabolic activity may act as markers of renewal, as indicated by their change in passaged cells [47]. While stem cells show few mitochondria in a perinuclear arrangement, senescent cells show scattered mitochondria throughout the cytoplasm (Figure 3). Further, undifferentiated neural stem cells show low ROS when compared to more differentiated cells, while generally differentiating cells show increased mass, increased ATP production, and reactive oxygen species (ROS) [6,47]. Overall, differentiation results in mitochondrial changes that include structure, morphology, localization, ATP levels, oxygen consumption, cellular respiration, and anaerobic ATP production [48].

Maintenance and repair of mitochondrial function is required throughout cellular life. However, our knowledge of mitochondria function in stem cells is limited [49]. What we do know is that mitochondria function is critical for energy production and protection against oxidative stress damage. Human ESC grows well in an environment of hypoxic conditions that maintains their pluripotency and resistance to spontaneous differentiation [47]. When considering stem cell manipulation and artificial cultivation, our limited knowledge of mitochondria energy states, biogenesis, dynamics, and degradation in stem cells present challenges of mitochondria vulnerability [49,50]. For example our understanding of mitochondrial status in iPS biology is rudimentary at best, and pose the question of how will a mature mitochondria function in a new “stem-like” state. Further, what affects will mitochondria age, nuclear synergy, mutations, and differentiation have on mitochondria function in manipulated and/or cultivated stem cells?

## 3. Conclusions

Identification and characterization of heterogeneity and/or variety of progenitors and their determinants is necessary for understanding their role in neuroprotection [7]. Cellular identification of neuronal progenitors with limited proliferative capacity subtypes and their specific stimuli is crucial for developing mechanistic based neuroprotective therapies that target the optimal cell type. To move forward in developing neuroprotectants in a calculated manner requires that we understand the stem cell players so that we can maximize therapies and minimize risks. Growing evidence indicates important links in stem cell function are mediated by factors of age, gender, telomerase, and mitochondria. These important and inter-related characteristics are poorly understood in all stem cell types. Our desire to develop neuroprotectants emphasizes a need to better understand these factors and how they influence stem cell renewal/neurogenesis. Increased understanding of these factors provides insight into the best approaches, applications, and targets for stem cell therapies. How these factors influence or deregulate functional pathways, can mediate more efficacious and novel neuroprotective targets.
